# Pheochromocytoma presenting as hypotension in a 12 year old female

**DOI:** 10.1186/1687-9856-2013-S1-P117

**Published:** 2013-10-03

**Authors:** Marichu P Mabulac, Lorna R Abad

**Affiliations:** 1 Section of Pediatric Endocrinology, Department of Pediatrics, University of the Philippines-Philippine General Hospital, Manila, Philippines

## Background

Pheochromocytoma is a rare cathecolamine tumor that presents with hypertension and the triad of headaches, palpitations, and sweating. We present a case with shock and tachycardia as the manifestation of pheochromocytoma.

## Case

12 year old female sought consult at the ER due to loss of consciouness. In the past, other symptoms included bouts of fatigue, headache, pallor, palpitations and profuse sweating of two years duration. The patient had history of body weakness, abdominal pain and vomiting associated with dyspnea hours prior to her admission. She had history of pheochromocytoma for which a left adrenal mass was excised three years prior her present admission. She then lost to follow-up after her operation. On clinical examination, the patient was drowsy, incoherent, diaphoretic, cyanotic, tachypneic (30 beats/min), tachycardic (200 beats/min) and no blood pressure appreciated. Her weight was 26 kg and height of 140 cm (-2SD), BMI: 13.2 (-2SD) Eye exam noted a grade III retinopathy. Initial blood work-ups showed anemia, hypoglycemia and hypocalcemia. Echocardiography showed concentric LV enlargement, systolic dysfunction, tricuspid regurgitation with LVEF of 31%. The CKMB was elevated but the troponin I was normal. Fluid resuscitation was initiated and intravenous inotropes agents were administered. Thereafter, her hemodynamic condition improved gradually and later found to have hypertensive episodes (range: from 120/100-180/120) even after discontinuing the inotropes. In view of hypertension and history of left adrenal tumor, work up for pheochromocytoma was done. Biochemical evaluation for pheochromocytoma revealed elevated 24-hour urine free metanephrines of 16.61mg (normal: 1mg/24hr). The computed tomography (CT) of the abdomen revealed a right suprarenal mass measuring 5.7 x 4.7 x 4.7cm. Patient was started on Terasozin and Carvedilol with normalization of blood pressure and resolution of other symptoms. She underwent laparoscopic mass resection. During tumor manipulation, the patient had several hypertensive crises (247/147mm Hg) which was treated with nitroglycerin and nicardipine drip. Post resection of the tumor, there was hypotension (60/40mmHg) which was given dopamine drip. The surgical specimen weighed 37.5grams, measured 5.5 x 4 x 2.3cm and histopathological examination confirmed the diagnosis of pheochromocytoma. At OPD, patient has no BP elevations and repeat 24 hour urine free metanephrines was normal at 0.06mg (normal: < 1mg/24 hour). Currently she is maintained with Hydrocortisone at (8mg/m2/day).

## Conclusion

This is a rare manifestation of pheochromocytoma and can be a challenge to the clinicians. Recurrent pheochromocytomas are unlikely in children but recurrent tumors may appear years after initial diagnosis.

